# Correlation of fundamental movement skills with health-related fitness elements in children and adolescents: A systematic review

**DOI:** 10.3389/fpubh.2023.1129258

**Published:** 2023-03-27

**Authors:** Cong Liu, Yuxian Cao, Zhijie Zhang, Rong Gao, Guofeng Qu

**Affiliations:** ^1^College of P.E. and Sports, Beijing Normal University, Beijing, China; ^2^Primary School Department, Tianjin Binhai Foreign Language School, Tianjin, China

**Keywords:** fundamental movement skills, flexibility, body composition, muscle strength and endurance, cardiopulmonary function, health-related fitness

## Abstract

**Objective:**

To examine the correlations between fundamental movement skills and health-related fitness elements (cardiopulmonary function, flexibility, body composition, muscle strength and endurance) in children and adolescents and investigate the evaluation methods and tools of fundamental movement skills and health-related fitness.

**Methods:**

Six electronic databases (Web of Science, PubMed, ProQuest, Scopus, EBSCO and CNKI) were searched, and the research literature on the correlation between children's and adolescents' fundamental movement skills and health-related fitness published since 2002 was collected. The guidelines of the Strengthening the Reporting of Observational Studies in Epidemiology (STROBE) statement and the Consolidated Standards of Reporting Trials (CONSORT) statement were used to evaluate the quality of the literature, and the sources, samples, measurement methods, main results and statistical data of the study were analyzed, summarized and discussed.

**Results:**

After applying the inclusion and exclusion criteria, 49 studies were included. There were 13 tools for evaluating fundamental movement skills and 4 tools for evaluating comprehensive health-related fitness in the included literature. Sufficient research evidence supports a significant positive correlation between fundamental movement skills and cardiopulmonary function (10, 100%) and muscle strength and endurance (12, 100%), and most studies support the positive correlation between fundamental movement skills and flexibility (4, 66.7%), and the significant negative correlation between fundamental movement skills and body composition (29, 67.4%). Studies used skinfold, AF%, BF%, FM, and FFMI as evaluation methods. They showed a consistently significant negative correlation between body composition and fundamental movement skills (9, 100%), while BMI or waist circumference as evaluation methods showed no consistent significant negative correlation result (20, 58.8%). Moreover, in the sub-item evaluation of fundamental movement skills, object manipulation, locomotor and balance skills were all significantly and positively correlated with cardiopulmonary function and muscle strength and endurance. In contrast, locomotor skills were more closely related to body composition than object manipulation skills.

**Conclusion:**

A significant correlation exists between children's and adolescents' fundamental movement skills and health-related fitness elements.

## 1. Introduction

Fundamental movement skills are the basis for more advanced and highly specific sports activities and are considered the “building blocks” of more advanced, complex movements required to participate in sports, games, or other context-specific physical activities (1, 2). Previous studies have confirmed that fundamental movement skills are associated with children's physical, cognitive, and social development and provide the basis for a positive and healthy lifestyle (3, 4). However, the relationship between fundamental movement skills and physical health in the current study has yet to be well documented, and whether fundamental movement skills improve the individual's physical health level needs a more detailed discussion.

Health-related fitness is closely related to health outcomes and health indicators (5), which has been defined by the President's Council on Physical Fitness as consisting of those specific elements of physical fitness that have a relationship with good health, including body composition, cardiopulmonary fitness (cardiopulmonary function), and musculoskeletal fitness (flexibility, muscle strength, and endurance) (6, 7). Evaluating health-related fitness elements can provide data that help formulate exercise prescriptions and establish reasonable and achievable fitness goals to motivate participants. Therefore, exploring the correlations between health-related fitness elements and fundamental movement skills will help us understand the role of fundamental movement skills in promoting physical health.

However, only a few studies have reviewed the association between fundamental movement skills and health-related fitness. In a review of fundamental movement skills and the health benefits of children and adolescents, Lubans et al. (8) found that fundamental movement skills were positively correlated with cardiopulmonary fitness (4 out of 4 studies) and negatively correlated with body weight (6 out of 9 studies). Nevertheless, the relationship between fundamental movement skills and musculoskeletal fitness has not been discussed due to the lack of relevant research at that time. In 2016, Cattuzzoa et al. (9) reviewed the association between motor competence and health-related fitness elements. They reported a positive association between motor competence and cardiorespiratory fitness and musculoskeletal fitness and an inverse association between body weight status. However, the motor competence mentioned in this review study cannot be equated with fundamental movement skills. Motor competence is a person's ability to execute different motor acts (10), including fundamental movement skills and motor coordination (11). Fundamental movement skills are often described more precisely as basic stability, object control and locomotor movements (2, 12, 13), while motor coordination is a general term that encompasses various aspects of movement competency (14), and needs the coordination of complex neural networks (15). Moreover, there are differences in the evaluation contents of motor competence and fundamental movement skills. Therefore, Cattuzzoa et al. (9) conclusion cannot be used to correlate fundamental movement skills and health-related fitness elements.

This review aims to systematically examine the correlations between fundamental movement skills and health-related fitness elements, investigate the evaluation methods and tools of fundamental movement skills and health-related fitness, and provide a scientific basis for theoretical and practical research on fundamental movement skills and health-related fitness.

## 2. Methods

### 2.1. Search of the literature

A structured electronic literature search was conducted under the Preferred Reporting Items for Systematic Reviews and Meta-Analyses (16). The search included six electronic databases (Web of Science, PubMed, ProQuest, Scopus, EBSCO, and CNKI). The retrieval was “[Title/Abstract] = (‘Fundamental Movement Skills' OR ‘Motor skill') AND [Title/Abstract] = (‘health related fitness' OR ‘health benefits' OR ‘body composition' OR ‘body mass index' OR ‘weight' OR ‘fat percentage' OR ‘cardiorespiratory fitness' OR ‘cardiopulmonary function' OR ‘musculoskeletal fitness' OR ‘muscle strength' OR ‘muscle endurance' OR ‘flexibility'). The search was conducted from January 1, 2000, to November 23, 2022, and only literature in English and Chinese published in peer-reviewed journals was considered.

Two researchers independently screened and reviewed the literature and jointly determined the final article list. If inconsistent screening results occurred, a third researcher was asked to decide.

### 2.2. Eligibility criteria

A PECO (population, exposure, comparison and outcome) approach (17) was used as inclusion criteria: (a) Population: participants were 3–16 years old and were in preschool education or school; (b) Exposure: fundamental movement skills, including comprehensive fundamental movement skills or subitems (locomotor, object manipulation, and balance skills); (c) Comparison: health-related fitness elements (cardiopulmonary function, muscle strength and endurance, flexibility, and body composition); (d) Outcome: report or data that makes it possible to estimate associations.

The exclusion criteria were as follows: (a) research articles on special groups, such as cardiovascular disease, developmental coordination disorders, mental disorders, etc.; (b) intervention study (literature on fundamental movement skills and health-related fitness association with an experimental intervention); (c) study sample of fewer than 50 people; (d) no cross-sectional data of fundamental movement skills and health-related fitness; (e) non-English or non-Chinese literature.

### 2.3. Data extraction and quality evaluation

The data extraction form retrieved the following information: first author and publication time, test method, health-related fitness elements, study design type, participant, and statistical method. Two researchers independently completed the data extraction, followed by discussion and cross-checking to ensure consistency and accuracy.

The literature quality was assessed using the guidelines of the Strengthening the Reporting of Observational Studies in Epidemiology (STROBE) statement (18) and the Consolidated Standards of Reporting Trials (CONSORT) statement (19), based on Lubans et al. (8) and Cattuzzoa et al. (9). The quality score for each study was based on six questions: (1) Did the study describe the participant eligibility criteria? (2) Were the participants randomly selected (or for the experimental studies, was the randomization process clearly described and adequately carried out)? (3) Did the study report the sources and details of the FMS assessment, and did the instruments have acceptable reliability for the specific age group? (4) Did the study report the sources and details of the assessment of potential benefits, and did all the methods have acceptable reliability? (5) Did the study report a power calculation, and was the study adequately powered to detect the hypothesized relationships? (6) Did the study report the numbers of individuals who completed each of the different measures, and did participants complete at least 80% of the FMS and benefit measures? The above questions were scored as 0 (missing or underdescribed) or 1 (clear description and presence), and the scores for all questions were combined. Studies scoring 0–2 were considered low-quality studies, studies scoring 3–4 were classified as medium-quality, and 5–6 were classified as high-quality.

## 3. Results

### 3.1. Basic information about the literature

**Figure 1:**
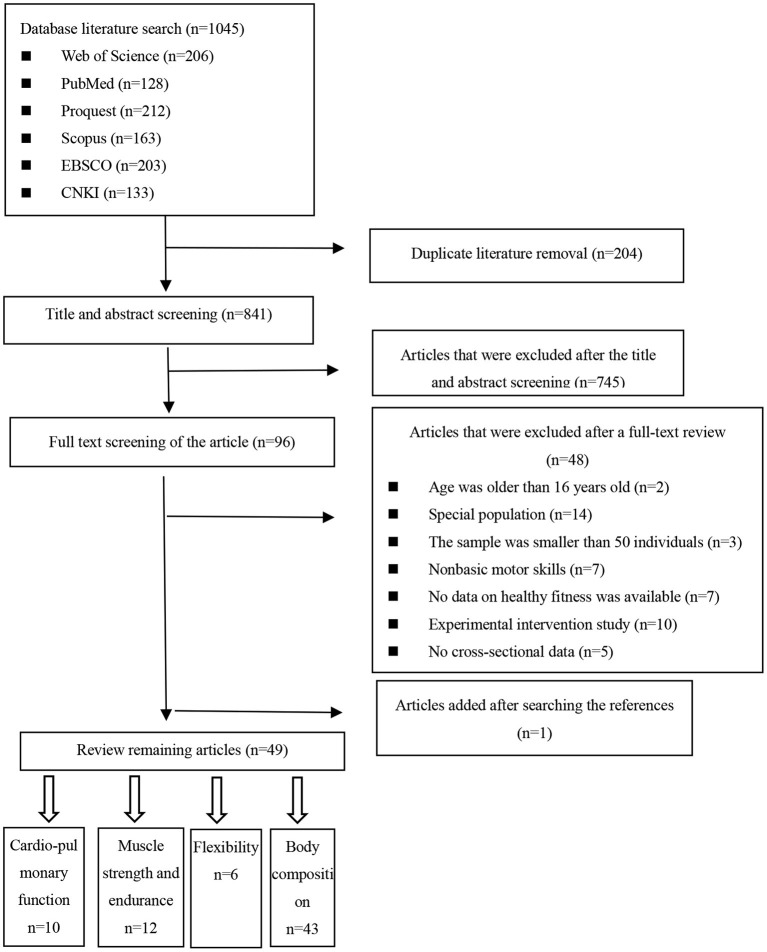
Flow chart of the literature review process.

Figure 1 shows the study selection flow chart. A total of 49 articles met the eligibility criteria. These articles all had cross-sectional data, including 44 cross-sectional studies, four longitudinal studies, and one long-term trend study. These articles were published from January 2002 to November 2022. There were 42 English studies and 7 Chinese studies. Eight of the studies were conducted in the UK, eight in China, six in the US, five in Australia, five in Iran, four in Finland, four in Ireland, two in Croatia, and one each in Canada, Slovenia, Brazil, Italy, the Czech Republic, South Korea and South Africa. The study participants ranged from 50 (20) to 6,917 (21). Details are given in Table 1.

**Table 1:** Summary of the included literature.

**Order**	**References**	**Date**	**FMS test method**	**Health-related fitness test method**	**Health-related fitness elements**	**Type of study**	**Sample**	**Statistical methods**
1	Aalizadeh et al. (22)	2013	TGMD-2	BMI	BC	C	241 (age 7–10)	Pearson correlation, multiple linear regressions
2	Behan et al. (23)	2022	TGMD-3, vertical jump, balance	BMI, WC, grip strength, plank, sit and reach, PACER,	BC, CVE, MF, Flex	C	2,098 (age 5–12)	MANCOVA
3	Bolger et al. (24)	2019	TGMD-2	BMI, WC, 550-meter walk/run	BC, CVE	C	296 (mean age 7.9 ± 2.0)	Pearson correlation
4	Bryant et al. (25)	2014	POC	BMI	BC	C	281 (age 6–11)	MANOVA
5	Bryant et al. (26)	2014	POC	BMI, BF%	BC	L	T1: 281 (mean age 8.9 ± 1.4); T2: 252 (mean age 9.8 ± 1.4)	Multiple linear regression
6	Butterfield et al. (27)	2002	TGMD	BMI	BC	C	65 (age 5–8)	Multiple linear regressions
7	Chen et al. (28)	2016	PE metrics	FitnessGram assessment	CVE, MF, Flex	C	565 (age 9–10)	Multiple linear regressions
8	Comeau et al. (29)	2017	Passport for Life, PLAYbasic	BMI, grip strength, PACER, BF%	BC, CVE, MF	C	145 (age 9–12)	Pearson correlation, Multiple linear regressions
9	Duncan et al. (30)	2017	POC	BMI, BF%	BC	C	248 (age 6–11)	MANCOVA
10	Duncan et al. (31)	2021	TGMD-2	BMI	BC	L	T1: 177 (mean age 4.0 ± 0.7); T2: 91 (mean age 5.0 ± 0.7)	Multiple linear regressions
11	Foulkes et al. (32)	2021	TGMD-2	BMI	BC	L	T1: 240 (mean age 4.5 ± 0.6); T2: 181 (mean age 10 ± 0.6)	Linear regression
12	Franjko et al. (33)	2012	TGMD-2, POLYGON	BMI, BF%	BC	C	73 (Grade 2)	Pearson's correlation
13	Gu et al. (34)	2021	PE Metrics	FitnessGram assessment, BMI	BC, CVE, MF	C	342 (mean age 8.4 ± 0.50)	Pearson correlation
14	Hardy et al. (21)	2012	POC	PACER, BMI	BC, CVE	C	6,917 (age 7–14)	Odds ratios (ORs)
15	Hua et al. (35)	2017	TGMD-2	BMI	BC	C	240 (age 7–10)	One-way ANOVA
16	Huan et al. (36)	2019	TGMD-3	NPFTSM-Preschool	MF, Flex	C	646 (age 3–6)	Canonical correlation coefficient
17	Hume et al. (37)	2008	Fundamental motor skills Assessment	BMI	BC	C	248 (age 9–12)	Pearson correlation
18	Huotari et al. (38)	2018	Move!	BMI	BC	S	T1: 2,390 (grade 9); T2: 1,346 (grade 9)	General linear model
19	Jaakkola et al. (39)	2019	Move!	PACER, curl-up test, push-up	CVE, MF	L	491; T1: mean age 11.26; T2: mean age 12.26	The standardized parameter estimates of the multigroup model
20	Jarvis et al. (40)	2018	POC	ALPHA, 20-meter sprint	BC, CVE, MF	C	*n =* 553 (age 9–12)	Zero order correlations
21	Jing et al. (41)	2019	TGMD-2	NPFTSM-Preschool	MF	C	498 (age 3–5)	Partial correlation and multiple regressions
22	Joensuu et al. (42)	2018	Move!	FMI, FFMI	BC	C	*n* = 594 (mean age 12.4 ± 1.3)	Unstandardized regression coefficients
23	Jones et al. (43)	2010	POC	BMI	BC	C	1,414 (age 9–11)	One-way ANOVA
24	Kelly et al. (44)	2019	TGMD-3	BMI	BC	C	*n* = 414 (age 6–12)	Independent samples t-tests
25	Kemp et al. (45)	2013	TGMD-2	Skinfolds, WC, BMI	BC	C	816 (mean age 6.87 ± 0.39)	One-way ANOVA
26	Khalaj and Amri (46)	2013	TGMD-2	BMI	BC	C	160 (age 4–8)	One-way ANOVA
27	Kim and Lee (47)	2016	TGMD-2	BMI	BC	C	216 (age 5–6)	Pearson correlation
28	Marinsek et al. (48)	2019	Eight FMS	BMI	BC	C	*n* = 322 (age 5–10)	MANCOVA
29	Morano et al. (49)	2011	TGMD-2	BMI	BC	C	80 (mean age 4.5 ± 0.5)	One-way ANOVA
30	Musalek et al. (50)	2017	MABC-2	Skinfolds, BMI	BC	C	152 (age 3–6)	Variance ANOVA
31	Nervik et al. (19)	2011	PDMS-2	BMI	BC	C	50 (age 3–5)	Pearson correlation, multiple regressions
32	Okely et al. (51)	2004	Fundamental motor skills Assessment	BMI, WC	BC	C	4,363 (grades 2, 4, 6, 8, 10)	Multiple linear regressions
33	Poulsen et al. (52)	2011	TGMD-2	BMI	BC	C	116 (mean age 8.6 ± 1.4)	Multiple linear regressions
34	Rainer and Jarvis (53)	2019	POC	ALPHA	BC, CVE, MF	C	307 (age 10–11)	Pearson product-moment correlation
35	Roberts et al. (54)	2012	MABC-2	BMI	BC	C	4,650 (age 4–6)	One-way ANOVA
36	Roscoe et al. (55)	2019	TGMD-2	BMI	BC	C	185 (age 3–4)	Univariate ANOVA
37	Shengkou et al. (56)	2015	TGMD-2	NPFTSM-Preschool, BMI	BC	C	289 (age 3–6)	Correlation analysis
38	Siahkouhian et al. (57)	2011	TGMD-2	BMI	BC	C	200 (age 7–8)	Pearson correlation
39	Slotte et al. (58)	2015	TGMD-2	BF%, AF%, WC, BMI	BC	C	304 (age 8)	Spearman's correlations
40	Spessato et al. (59)	2012	TGMD-2	BMI	BC	C	178 (age 4–7)	Multiple regression
41	Vameghi et al. (60)	2013	OSU-SIGMA	BMI	BC	C	400 (age 4–6)	Multiple regression
42	Vameghi et al. (61)	2013	OSU-SIGMA	BMI	BC	C	600 (age 3–6)	Kendall's tau-b test
43	Webster et al. (62)	2021	TGMD-2	BMI, BF%, FMI, FFMI	BC	C	244 (mean age 6.05 ± 2.01)	Multiple linear regressions
44	Wesley et al. (63)	2016	TGMD-2	BMI	BC	C	85 (mean age 12.7 ± 0.4)	Multiple linear regressions
45	Yameng et al. (64)	2019	TGMD-3	NPFTSM-Preschool, BMI	MF, Flex, BC	C	201 (age 3–5)	One-way ANOVA
46	Yang et al. (65)	2015	TGMD-2	BMI	BC	C	1,200 (age 3–7)	One-way ANOVA
47	Yanmin et al. (66)	2021	TGMD-3	NPFTSM-Preschool	MF, Flex	C	304 (age 3–6)	Partial and bivariate correlation, multiple regressions
48	Yuanchun et al. (67)	2013	TGMD-2	BMI	BC	C	852 (age 6–9)	One-way ANOVA, Kendall's tau-b test
49	Zuvela and Kezic Krstulovic (68)	2016	POLYGON	standing long jump, sit and reach, 1/4-Mile	MF, CVE, Flex	C	90 (age 8)	Multiple linear regressions

FMS, fundamental movement skills; TGMD, test of gross motor development; POC, get-skilled get-active process-oriented checklists; POLYGON, a new fundamental movement skills test for 8-year-old children; PE metrics, physical education metrics elementary assessments; Move!, monitoring system for physical functional capacity; MABC, movement assessment battery for children-second edition; PDMS, peabody developmental motor scales; OSU-SIGMA, the Ohio State University scale of intra gross motor assessment; BMI, body mass index; WC, waist circumference; PACER, progressive aerobic cardiovascular endurance run; BF%, body fat percentage; FitnessGram Assessment, Presidential Youth Fitness Program assessment; ALPHA, the health-related fitness test battery for children and adolescents; NPFTSM-Preschool, National Physical Fitness Test Standards Manual-Preschool part; AF%, abdominal fat percentage; FMI, fat mass index; FFMI, fat-free mass index; BC, body composition; CVE, cardiopulmonary function; MF, muscle strength and endurance; Flex, flexibility; C, cross-sectional study; L, longitudinal study; S, secular trend study;T1, the first measurement; T2, the second measurement; MANCOVA, multivariate analysis of covariance; ANOVA, analysis of variance.

### 3.2. Quality evaluation of the research literature

The authors had a 94% consensus on the study assessment criteria and reached a complete consensus after discussion. Twenty-eight studies were identified as high-quality, 21 were rated as moderate-quality, and none were classified as low-quality. Most studies used valid and reliable measures of fundamental movement skills assessment. All studies reported reliable data on their potential benefits, methods for valid calculations, and whether the study had sufficient evidence to support the hypothesis relationship.

### 3.3. Fundamental movement skills assessment tools

The literature included 13 fundamental movement skill assessment tools, as shown in Table 2. Because the research topics were limited to fundamental movement skills, the literature using Koperkoordination-Test fur Kinder (KTK) (81) and Bruininks-Oseretsky Test of Motor Proficiency (BOTMP & BOT-2) (82), mainly used to test motor coordination and fine motor skills, was omitted.

**Table 2:** List of fundamental movement skill evaluation tools.

**Evaluation tools**	**Full name**	**Publisher, date**	**Applicable age**
TGMD	Test of gross motor development	Ulrich (69)	3–10
TGMD-2	Test of Gross Motor Development-2	Ulrich (70)	3–10
TGMD-3	Test of Gross Motor Development-3	Ulrich (71)	3–10
POC	Get-skilled Get-active process-oriented checklists	Bibby (72)	3–12
Fundamental motor skills assessment	Fundamental motor skills assessment: A manual for classroom teachers	Walkley et al. (73)	3–10
POLYGON	A new fundamental movement skills test for 8-year-old children	Zuvela et al. (74)	8
OSU-SIGMA	The Ohio State University scale of intra gross motor assessment	Loovis and Ersing (75)	2.5–14
MABC-2	Movement assessment battery for children-2	Henderson and Sugden (10)	3–16
PE metrics	Physical education metrics elementary assessments	Dyson et al. (76)	3–18
PLAYbasic	The physical literacy assessment for youth	Kriellaars (77)	7–12
Passport for life	Passport for life assessment	Physical & Health Education Canada (78)	8–12
PDMS-2	Peabody developmental motor scales 2nd edition	Folio and Fewell (79)	0–6
Move!	Monitoring system for physical functional capacity	The Finnish National Ministry of Education and Culture (80)	7–18

The included studies have differences in the selection of fundamental movement skills evaluation tools. Thirty-five studies (71.5%) used process assessment, including 20 studies that used TGMD-2 as a single test, one used TGMD, 7 used POC, 3 used the Fundamental motor skills Assessment, two used OSU-SIGMA, 1 used PDMS-2, and one used Passport for Life and PLAYbasic. Eight (16.3%) studies used outcome assessments, 2 used the PE metric, 3 used the Move!, 1 study used POLYGON, and 2 used MABC-2. Six (12.2%) studies used a combination of process and outcome assessment, 5 of which used the TGMD-3 assessment tool, and 1 used the TGMD-2 and POLYGON.

### 3.4. Health-related fitness assessment tools

Among the included literature, there were 4 tools for comprehensive evaluation of health-related fitness, including the health-related fitness test battery for children and adolescents (ALPHA), FitnessGram assessment (FitnessGram), Monitoring system for physical functional capacity (Move!) and National Physical Fitness Test Standards Manual-Preschool part (NPFTSM-Preschool).

ALPHA was published by Ruiz et al. (83) based on assessment methodology for physical activity levels in the European Member States. Testing included a 20-meter shuttle run, grip strength, standing long jump, body mass index, skinfold thickness and waist circumference.

FitnessGram is now the educational assessment of the Presidential Youth Fitness Program (84). The test items include pull-ups for boys/modified pull-ups for girls, straight leg sit-ups, shuttle run, standing broad (long) jump, 50-yd dash, and softball throw for distance, 600-yd run/walk, and three aquatic tests that are rarely used.

Move! is a national physical functional capacity monitoring and feedback system for Finnish 5th and 8th-grade pupils (80). Move! consists of eight sections of measurements that provide information about the state of physical functional capacity. The sections measure pupils' endurance, strength, speed, mobility, balance, and fundamental movement skills. The specific items are the 20-meter line run, five continuous jumps, upper body lift, push-up, body mobility, squat, lower back extension, right and left shoulders, and a throw-catch combination.

NPFTSM-Preschool (85) was promulgated by the Ministry of Education of the People's Republic of China in 2003. The test content included morphometric measures, a 10-meter return run, standing long jump, tennis throwing, continuous jump, sit and reach, and walking on the balance beam.

In addition to the above tools, some studies selected different evaluation tools for comprehensive utilization; for example, Behan et al. (23) adopted BMI and waist circumference, sit and reach, grip strength (86), plank (87), a 20 m sprint run (88) as health-related fitness assessment content.

Moreover, health-related fitness assessment can be an overall assessment of all elements, and it can also be an assessment of only one element. In evaluating sub-elements of health-related fitness, various tools have been used in the included studies.

The cardiopulmonary function was assessed by the PACER (Progressive Aerobic Cardiovascular Endurance Run), 550 m walking and running test (89) or long-distance running (68, 89, 90).

The musculoskeletal function was evaluated by two groups, muscle strength and endurance, and flexibility. Methods to assess muscle strength and endurance included grip strength, curl-ups, push-ups, plank, and standing long jump. Furthermore, methods to assess flexibility included sit and reach and trunk lifting.

Body composition assessment methods were body mass index (BMI), waist circumference (WC), skinfold thickness, body fat percentage (BF%), abdominal fat percentage (AF%), fat mass index (FMI), and fat-free mass index (FFMI).

### 3.5. Correlation between fundamental movement skills and cardiopulmonary function

A total of 10 articles (19, 23, 24, 28, 29, 34, 39, 40, 53, 68) have studied the correlation between fundamental movement skills and cardiopulmonary function, and all the findings showed a significant positive association between fundamental movement skills and cardiopulmonary function.

Among the subitems of fundamental movement skills, five studies (19, 23, 29, 34, 39) showed a significant correlation between locomotor skills and cardiopulmonary function, six studies (19, 23, 28, 29, 34, 39) showed a significant correlation between object manipulation skills and cardiopulmonary function, and three studies (19, 29, 39) showed a significant association between balance skills and cardiopulmonary function. Thus, there is strong evidence for a positive association between the total and subitems of fundamental movement skills and cardiopulmonary function.

### 3.6. Correlation between fundamental movement skills and muscle strength and endurance

A total of 12 studies (23, 28, 29, 34, 36, 39–41, 53, 64, 66, 68) were included that evaluated the correlation between fundamental movement skills and muscle strength and endurance. These studies found a significant positive correlation between total fundamental movement skills and muscle strength and endurance.

Regarding the subitems of fundamental movement skills, seven studies (23, 29, 34, 36, 39, 64, 66) showed a positive correlation between locomotor skills and muscle strength and endurance, eight studies (23, 28, 29, 34, 36, 39, 64, 66) showed a significant correlation of object manipulation skills with muscle strength and endurance, and three studies (34, 40, 68) showed a significant correlation between balance skills and muscle strength and endurance. All studies supported the conclusion that the total and subitems of fundamental movement skills were significantly positively correlated with muscle strength and endurance.

### 3.7. Correlation between fundamental movement skills and flexibility

Six studies examined the correlation between fundamental movement skills and flexibility. Four studies (23, 28, 36, 66) showed a significant correlation between fundamental movement skills and flexibility, and 2 showed no significant correlation (29, 64).

Moreover, as to the subitems of fundamental movement skills, one study showed that locomotor skills were significantly associated with flexibility, but the correlations between object manipulation skills and flexibility were not significant (66). Another study (23) found that three subitem skills in the 9–10 age group and the flexibility association were significant; however, in the 11–12 age group, locomotor, and balance skills were still significant, while object manipulation skills were no longer significant. These results indicate that the evidence of the relationship between the total and subitems of fundamental movement skills and flexibility is uncertain.

### 3.8. Correlation between fundamental movement skills and body composition

Forty-three studies examined the correlation between fundamental movement skills and body composition. Twenty-nine studies showed a significant association of overall fundamental movement skills with body composition, and 14 showed no significant association with body composition. Of the 29 significantly related studies, 18 studies used BMI alone as an assessment, four studies (26, 29, 31, 33) used BMI and BF, two studies (24, 53) used BMI and waist circumference, one study (50) used BMI and skinfold thickness, one study (45) used BMI, waist circumference and skinfold thickness, 1 used BF%, AF%, BMI, and waist circumference (58), 1 used FMI and FFM (42), and 1 used BF%, BMI, FMI, and FFMI (62). Of the 14 studies showing no significant association with body composition, 13 studies (19, 22, 27, 35, 37, 40, 47, 53, 55, 56, 59, 64, 67) used BMI as a single body composition assessment method, and one article (24) used both waist circumference and BMI. Overall, studies that used skinfold, AF%, BF%, FM, and FFMI as evaluation methods obtained consistently significant negative correlation results, while in studies that used BMI or waist circumference as evaluation criteria, there was no consistent significant correlation result.

Furthermore, as to the subitems of fundamental movement skills, three studies (23, 29, 39) showed a consistent inverse correlation of balance skills with body composition. Meanwhile, locomotor skills and body composition reflected a more significant correlation than object manipulation skills. Six studies (34, 35, 45, 54, 57, 62) showed that object manipulation skills were not associated with body composition, while locomotor skills were significantly associated with body composition. Therefore, there is evidence that the relationship between locomotor skills and body composition is closer than that of object manipulation skills.

## 4. Discussion

The main objective of this review was to explore the correlation between fundamental movement skills and health-related fitness elements in children and adolescents. We found strong evidence from cross-sectional study results that the children's and adolescents' fundamental movement skills and cardiopulmonary function, muscle strength and endurance had a significant positive correlation. These results complement the need for correlation analysis between fundamental movement skills and musculoskeletal function by Lubans et al. (8) and also make up for the lack of specific correlation analysis between fundamental movement skills and health-related fitness in Cattuzzoa et al. (9).

The positive correlation between fundamental movement skills and cardiopulmonary function may be related to the role of fundamental movement skills in promoting physical activity. Previous studies have proven that fundamental movement skills are associated with moderate- to high-intensity physical activity (4, 24, 25, 29). Bolger et al. (24) believed that people with higher fundamental movement skills are more likely to participate in organized physical activities, which will allow them to obtain more guidance on basic athletic skills from coaches and promote the improvement of their physical activity intensity.

As for the positive correlation between fundamental movement skills and muscle strength and endurance, this may be because fundamental movement skills contribute to the maturation of skeletal and neuromuscular. Freitas et al. (91) believed that individual differences in fundamental movement skills interact with the habits of play and physical activities, as well as with the maturation of children's bones and neuromuscular. Stodden et al. (92) also noted that fundamental movement skills require the generation and decay of physical strength, which is related to the strength of the muscle itself and the neural function related to muscle movement.

The negative correlation of fundamental movement skills with body composition has been confirmed in most studies but has yet to obtain consistent results, which may be related to how body composition is assessed. Using BMI and waist circumference as evaluation criteria did not obtain consistent correlation results, while studies with skinfold, BF%, AF%, FM, FM, and FFMI as evaluation results showed consistent negative correlation results. Previous studies have also found that BMI and waist circumference are proxy measures and should not be considered accurate measures of total body or abdominal fat (26, 93, 94). In assessing body composition, it is crucial to assess weight status using more accurate methods than BMI alone to obtain more precise evidence.

A possible reason for the negative correlation of fundamental movement skills with body composition is that an increased amount of body fat hinders the performance of fundamental movement skills (9), which may affect the control of posture. Marinsek et al. (50) found that overweight boys did not lean slightly forwards during running compared with non-overweight boys, did not bend their hips and knees during dribbling, and did not side to the target during single-handed hitting. From the perspective of postural control, strengthening the proficiency of motor skills or increasing the muscle strength of body control can reduce the adverse effects of body weight. Based on this, when teaching exercises to obese students, more attention should be given to the exercise of movement and posture control, such as strengthening the muscles and training fundamental movements.

Most studies support a significant positive correlation between fundamental movement skills and flexibility, but the association of fundamental movement skills with flexibility still needs further study. Indeed, developing flexibility is very important for adolescent health, but there is insufficient evidence that flexibility is directly related to individual health status (90), which could be related to the limitations of flexibility assessment. Flexibility mainly reflects the stretching and elasticity of the joints, ligaments and muscles. Excessive tension or relaxation can affect the performance of movement skills (95). Studies have found that children with low exercise ability have heterogeneous fitness characteristics, and an extreme range of flexibility and inflexibility can be observed in these children (9). However, the current commonly used flexibility assessment method (sit and reach) cannot detect a lack of function due to muscle relaxation. Of the studies on flexibility assessment included in this review, one used trunk lifting to assess flexibility (28), which showed that fundamental movement skills were significantly associated with flexibility. However, the use of trunk lifting has a specific need for trunk muscle strength and endurance, and there is a lack of validated methods for evaluating the flexibility of children and the elderly (90). Overall, an appropriate level of flexibility has positive implications for motor skill development and physical health, but exploring scientific and reasonable methods of flexibility assessment should receive more attention.

In addition, this study found some similarities and differences in the correlations between the fundamental movement skills sub-item (locomotor, object manipulation and balance skills) and health-related fitness elements. Locomotor, object manipulation and balance skills with cardiopulmonary function, muscle strength and endurance presented consistent positive correlations, while locomotor and object manipulation skills were associated differently with body composition. Six studies showed that object manipulation skills were not associated with body composition, while locomotor skills were significantly associated with body composition; this is quite different from the conclusions of some previous studies, in which object manipulation skills were given great attention. Barnett et al. (96) noted that the relationship between object manipulation skills and physical activity is seen as a “positive feedback loop” and that those with better object manipulation skills may be more willing to participate in activities involving these skills. Vlahov et al. (97) also found that object manipulation skills in a prospective study of preschool children were better predictors of health-related fitness. However, the health-promoting effect of object manipulation skills on health-related fitness is more of a concern for the individual's “willingness to participate.” There may be great obstacles between “willingness to participate” in physical activities and health-related fitness, such as the impact of the sports environment and atmosphere, the shift of physical entertainment to internet entertainment, and the compromise between students' physical health goals and the goals of school knowledge acquisition.

Conversely, developing individual locomotor skills is often associated with greater body calorie expenditure, which may contribute to maintaining a healthy body weight. Okely et al. (98) noted that locomotor skills in overweight children tend to be more difficult to show because they need more support and have a greater obstacle to exercise than object manipulation skills. Locomotor skills can better promote the maintenance of healthy body weight in the early stage of individual movement development, which has the same positive significance as promoting object manipulation skills to encourage participation in physical activity.

### 4.1. Limitations and suggestions for future research

Our study has limitations. Due to the lack of longitudinal research literature, this study only analyzed cross-sectional outcomes. Due to the various evaluation tools and large differences in the outcome data types of the reviewed articles, this review does not offer a quantitative summary (i.e., meta-analysis). With the increase in the research literature, future reviews can analyze the impact of fundamental movement skills on health-related fitness from a longitudinal perspective, explore scientific teaching strategies of fundamental movement skills, and conduct quantitative research data analysis to obtain more accurate correlations.

## 5. Conclusion

This systematic review found strong evidence that fundamental movement skills correlated with health-related fitness elements (cardiopulmonary function, muscle strength and endurance, and body composition) in children and adolescents. Most of the studies supported the conclusion that fundamental movement skills were also positively correlated with flexibility. In the fundamental movement skills subitems, object manipulation, locomotor, and balance skills were significantly and positively correlated with cardiopulmonary function and muscle strength and endurance, while locomotor skills were more closely related to body composition than object manipulation skills.

## Author contributions

CL and RG participated in the study design and protocol and wrote the manuscript. GQ sorted out the research process and retrieved literature. YC and ZZ screened the literature and drafted the manuscript. All authors reviewed the manuscript.
